# Effects of heat stress and dehydration on cognitive function in elite female field hockey players

**DOI:** 10.1186/s13102-018-0101-9

**Published:** 2018-06-19

**Authors:** Hannah MacLeod, Simon Cooper, Stephan Bandelow, Rachel Malcolm, Caroline Sunderland

**Affiliations:** 1 Sport, Health and Performance Enhancement (SHAPE) Research Centre, Department of Sports Science, School of Science and Technology, Nottingham Trent University, Nottingham, England NG11 8NS UK; 2 School of Sport, Exercise and Health Sciences, Loughborough University, Loughborough, England UK

**Keywords:** Decision-making, Skill, Intermittent exercise, Team sports, Cognitive performance, Hypohydration

## Abstract

**Background:**

It has previously been suggested that heat exposure and hypohydration have negative effects on cognitive performance, which may impact upon sporting performance. The aim of the present study was to examine the independent effects of heat stress and hypohydration on cognitive performance in elite female field hockey players.

**Methods:**

Eight unacclimatised elite field hockey players (age: 22 ± 3 y; height: 1.68 ± 0.05 m; body mass: 63.1 ± 6.0 kg) completed a cognitive test battery before and after 50 min of field hockey specific exercise on a treadmill in four experimental trials; two in hot conditions (33.3 ± 0.1 °C), and two in moderate (16.0 ± 3.0 °C), both with and without ad libitum water intake.

**Results:**

On the visual search test, participants were faster overall in the heat (1941 vs. 2104 ms, *p* = 0.001). Response times were quicker in the heat on the Sternberg paradigm (463 vs. 473 ms, *p* = 0.024) and accuracy was improved (by 1.9%, *p* = 0.004). There was no effect of hydration status on any of the markers of cognitive function.

**Conclusions:**

Overall, the findings suggest that in elite field hockey players exposure to heat enhances response times and/or accuracy on a battery of cognitive function tests. However, hypohydration does not appear to affect cognitive performance in elite field hockey players.

## Background

Field hockey demands an extensive requirement for high levels of mental processing to successfully perform in a dynamic environment. In addition, successful skilled performance has a substantial cognitive requirement [1], which may be susceptible to a number of physiological and environmental factors [2], for example heat, hydration, exercise and their interactions. This is of particular interest in team sports, where players are commonly subjected to hot and humid conditions (e.g. Olympic Games, Beijing 2008, Rio 2016, Football World Cup Qatar 2022) and the resulting hypohydration, which can reach body mass losses in excess of 2% by the end of match-play [3]. Findings suggest that field hockey skill performance in the heat is negatively influenced by hypohydration, as a result of both an increase in the number of errors and an increase in decision-making time [4].

The negative effect of heat stress on physical performance is well documented [5–12]. However, there is a degree of conjecture surrounding its influence on cognitive function, primarily due to a lack of systematic research. Most studies report a cognitive function decrement in the heat [6, 13–16], but some report no effect [17, 18], while others report an improvement in cognitive function [19]. During exposure to hot environments, decreases in cognitive function have been reported when increases in core body temperature were beyond compensatory levels [20, 21]. It is apparent that heat stress may compromise cognitive performance with the severity of the degradation dependent on the rise in core temperature, skin temperature, interplay between core and skin temperature and the complexity of the task [2, 22]. The increased demand for attention and effort involved in complex tasks could contribute to the negative influence of heat stress [2], with the interplay between a number of psycho-physiological pathways suggested to influence the changes in cognitive function [2, 23].

In much of the literature to date examining the effects of hydration status on cognition, exercise in the heat has been used as the dehydration protocol. Such studies typically results in hypohydration of ~ 2% body mass loss and have demonstrated decrements in various cognitive functions, including working memory, vigilance and perception [24–28]. However, some studies have reported no effect of exercise induced hypohydration on short-term memory, concentration and choice reaction time [29–31]. The interpretation of previous findings is however difficult due to differences between the participant characteristics (sex, training level, training status, acclimation status), the exercise protocol (duration, intensity, anaerobic exercise, aerobic exercise), and the cognitive tests employed [32]. For example, cognitive tests can be split into complex or simple tasks, where the literature indicates that complex tasks are more susceptible to environmental stressors [2, 22, 23, 33], whilst simpler tasks are unaffected by such stressors. Determining whether a task is simple or complex is problematic however, as different brain regions may be activated and repeated completion of a cognitive test may change it from complex to simple for an individual [2].

The interaction of exercise further complicates the potential influence of hypohydration and heat stress on cognitive performance. It has been hypothesised that the increase in metabolic load associated with exercise, as demonstrated by an elevation in heart rate and core temperature, increases arousal levels, which in turn improves cognitive performance [34]. Improvements in cognitive performance following exercise appear to be made as a result of faster response times with no increases in incorrect responses [35–38]. High-intensity bouts of exercise may facilitate a narrowing of attention to block out any irrelevant cues and improve focus on task-relevant information [39]. However, when the exercise intensity or duration is sufficient to bring about symptoms of central and/or peripheral fatigue, along with the associated hormonal changes, cognitive performance may decline [34]. A recent meta-analysis highlighted that moderators such as exercise duration, intensity, cognitive test and fitness are key in examining the influence which exercise has on cognitive function [40]. However, more recently it has been suggested that high intensity exercise, as experienced in team games, has a positive influence on executive function regardless of fitness level [41]. Two studies to date have examined the effects of games type activity, specifically simulations of the activity of goal line officials and soccer referees, in the heat on cognitive function [42, 43]. In both cases, no differences in cognitive function (vigilance and dual task) between temperate and hot environments were reported. The goal line official protocol incorporated small side-stepping movements which maintained core temperature at 37.1 °C and the referees protocol (based on Drust et al. [44]) elicited a mean core temperature of ~ 38.5 °C at the end of exercise. The authors suggested that the internal load of the protocol may not be reflective of soccer and thus not sufficient to have altered cognitive function [42]. Therefore, the effect of team sport activity in the heat on cognitive function warrants further investigation.

It has been suggested that exercise-induced dehydration, that may occur during competitions in hot environments, may impair complex cognitive tasks that require an additional resource allocation to a greater extent than simple, autonomous tasks due to the notion of allocated resources [31, 45]. These findings are particularly noteworthy due to the extensive environmental, physiological and cognitive demands placed on field hockey players and team sport athletes in general, which may increase the attentional resources required for optimal cognitive performance. Therefore, their combined effects warrant further investigation.

As a result of the conflicting evidence regarding the independent and differing influences of exercise, heat and dehydration on cognitive function, it remains unclear whether the complex relationship between these stressors may increase the potential for cognitive performance to be impaired above that associated with each individual stressor. To our knowledge, the interaction between sport-specific intermittent exercise in the heat and moderate dehydration on cognitive performance has not previously been investigated. Therefore, the aim of the present study was to determine the effect of 2% dehydration and heat stress on cognitive performance in elite female field hockey players following field hockey-specific intermittent exercise.

## Methods

### Participants

Eight healthy unacclimatized elite female field hockey players volunteered for the study. Six participants had normal menstrual cycles, and two had been taking oral contraceptives for more than 1 yr. The mean (± SD) age, body mass (BM), height, and maximal oxygen uptake (VO_2_ max) of the participants were 22 ± 3 yr., 63.1 ± 6.0 kg, 167.5 ± 5.3 cm, 53.4 ± 2.2 ml^.^kg^-1.^min^− 1^ respectively. All participants were outfield players involved in regular training. After completion of a health screen questionnaire, all participants provided their written informed consent for this study, which was approved by Nottingham Trent University’s Ethical Advisory Committee (reference number: xxxviii).

### Experimental design

Each participant completed four experimental sessions; two sessions in hot environmental conditions (33.3 ± 0.1 °C, 59 ± 1% rh), with and without fluid intake (HF, HNF), and two sessions in moderate environmental conditions (16 ± 3 °C, 53 ± 2% rh), with and without fluid (MF, MNF). 33 °C was chosen for the hot environments as this temperature is representative of that expected for the Olympics in Tokyo 2020. Experimental trials were conducted in a randomised order.

### Familiarisation

Participants reported to the laboratory on three separate occasions prior to the main trials. Session one involved measurements of height and body mass, and an incremental treadmill test to determine maximal oxygen uptake (VO_2_ max). Participants completed a submaximal test consisting of 4–6, 3 min stages, whereby speed was increased every 3 min on a 1% gradient, until lactate threshold was achieved. Lactate threshold was defined as the fastest speed with less than a 1 mmol.l^− 1^ increase in blood lactate concentration above the preceding levels. After a brief interval (~ 10 min) participants exercised at the speed immediately preceding the lactate threshold with an increase in the gradient of 1% per minute [46]. Each participant ran to volitional exhaustion and achieved at least two out of four criteria for attaining VO_2_ max [47]. The 4 criteria for VO_2_ max were: 1) a plateau in oxygen uptake; 2) respiratory exchange ratio > 1.1; 3) high blood lactate concentration and 4) heart rate > 90% of age predicted maximal. Sessions two and three involved familiarisation with the computer-based cognitive test battery (~ 15 min; CV: 4.3–6.7%; [48]) and the intermittent treadmill protocol, in both moderate (16 °C, 50% relative humidity [rh]) and hot (33 °C, 60% rh) conditions (CV: 0.6–3.5%; [49]). Sweat rate was calculated in both familiarisation sessions to establish the duration of passive hyperthermia required in the proceeding trials.

### Experimental procedures

All trials were conducted during the follicular phase of the menstrual cycle (days 4–11) to control for changes in basal body temperature and modifications to fluid regulatory hormones. A minimum of 5 days elapsed between trials to ensure adequate recovery. Participants were able to rehydrate by drinking water ad libitum to try to maintain euhydration. Participants received no fluid during the dehydration trials.

Participants recorded their diets during the 48 h prior to their first experimental trial and this was replicated prior to the remaining trials to control for nutritional status. Participants abstained from intense exercise, alcohol and caffeine for 48 h prior to each trial and were instructed to drink at least 2 L of water per day to ensure participants arrived at the laboratory in a state of euhydration. All trials were conducted at the same time of day (08.00 h − 12.00 h) to control for circadian influences. The clothing worn by the participants consisted of a t-shirt, shorts, socks and trainers; the same attire was worn for all trials.

### Field hockey specific intermittent treadmill protocol

The FHITP used for the main trials consisted of 50 min of activity, divided into 2 × 25 min blocks of exercise separated by a 10 min intermission designed to replicate half time [49]. The protocol was performed on a motorised treadmill (h/p/cosmos Pulsar 4.0, Nussdorf-Traunstein, Germany) housed in an environmental chamber (WIR52-20HS, Design Environmental Ltd., Gwent, Wales, U.K) and consisted of different exercise intensities observed in match-play [50, 51]. The activities were standing, walking, fast walking, jogging, cruising and lunging. To individualise the protocol, time spent jogging and cruising were set at a pace equivalent to 75% (11 ± 2 km.h^− 1^) and 95% (14 ± 2 km.h^− 1^) VO_2_ max, which is reflective of speeds in hockey [50, 51] and other team sport simulations [52]. The total distance covered is ~ 7000 m, which is representative of distances covered in matches [51], and the movement activities had a change in speed occurring every 8 s on average. In addition, lunging movements were included as this is integral to field hockey and is an eccentric intense exercise. A detailed discussion relating to the FHITP and how it reflects the demands of a field-hockey match is available in MacLeod and Sunderland [49]. Participants were required to hold a hockey stick for the entire duration of the protocol. The treadmill gradient was set at 1% to reflect the energy cost of outdoor running.

### Cognitive test battery

Participants were asked to complete a cognitive test battery that lasted for approximately 15 min. This cognitive battery has previously been employed in similar research, to determine the effect of soccer match-play, in hot environmental conditions, on cognitive performance [53]. All cognitive tests were delivered from a single software package on laptop computers with millisecond-resolution timing. Participants completed two full familiarisations sessions of the cognitive test battery prior to the first experimental trial which has previously been shown to minimise learning effects (CV: 4.3–6.7%; [48]). Furthermore, each test (and each test level) was preceded by 3–6 practice stimuli, for which feedback was provided regarding whether the participants response was correct or not (no feedback was provided once the tests started). Data for these practice stimuli were discarded. The cognitive function tests were always administered in the following order:

### Stroop test

This test measures the sensitivity to interference and the ability to suppress an automated response (time needed to read the colour words rather than the time it takes to name the colour of the letters) [54]. The baseline level contained 15 stimuli (reading colour names printed in white on a black background), the colour-interference level (naming the font colour rather than reading the printed colour name, which was always incongruent) comprised 40 stimuli. Each colour word was placed on the centre of the screen with the target and distractor presented at random on the left or right side of the stimulus word, with target position counterbalanced for left and right side within each test level. Participants were instructed to press the left or right arrow key as quickly as possible to indicate the position of the target word. Response times and the percentage of correct responses were recorded. To avoid undue influence of unusually slow or fast responses (outliers) on the analysis, response times were filtered, removing times faster than 100 ms for both test levels, and RTs slower than 1300 ms and 2000 ms for the baseline and colour-incongruent level respectively.

### Visual search

The visual search (VS) test was used to assess visuo-motor response times and comprised two levels, baseline and complex. On both levels, participants were instructed to press a key as soon as the participant could detect a triangle on the screen. After each response, new targets appeared with random delays of at least 500 ms. Response times and the percentage of correct responses were recorded. A very similar version of this test was previously found to be sensitive to the effects of prolonged exercise [55].

The baseline level contained 20 targets, which were drawn in solid green lines on a black background. In the 40 complex level targets, moving random dots covering the entire screen served as background distractors. New target triangles were initially drawn with just a few visible dots of each line, and the density of these points increased linearly with time until a key press response was registered. The screen was re-drawn every 250 ms with a new set of distractor dots, inducing the distracting visual effect of a flickering background. The baseline level is designed to assess simple visuo-motor speed, whereas the complex level introduces an additional complex visual processing component response times were filtered < 300 ms and > 850 ms for the simple and > 6000 ms for the complex level.

### Sternberg working memory test

The Sternberg test [56] is used to assess serial working memory scanning function. The test employed 3 working memory loads; 1, 3 and 5 items, which had to be held in working memory for correct performance. The baseline 1-item task, for which the target was always the number ‘3’, is a measure of basic information processing speed. For the 3 and 5 item levels, the targets were a list of 3 and 5 letters, respectively.

For all working memory loads, the target item (s) were displayed together with on-screen instructions that asked participants to press the right arrow key if the participant thought any of the following choice items was a target item, and to press the left arrow key otherwise. Sixteen choices were presented for the 1 item level, and 32 choices for the 3 and 5 item levels. Correct choices were counterbalanced between both responses keys. Response times (between 100 ms and 1500 ms) and the percentage of correct responses were recorded.

### Main trials

Participants arrived at the laboratory ~ 40 min before the commencement of each trial, after an overnight fast (> 10 h), other than the ingestion of 500 ml of water ~ 90 min previously. A urine sample was voided for the determination of urine osmolality (*U*_osm_) to verify hydration status. The trial was abandoned and rescheduled if the participant was dehydrated (*U*_osm_ > 900 mosmol.kg^− 1^). Nude body mass was recorded using calibrated digital scales, (Seca 873, Hamburg, Germany) accurate to the nearest 0.05 kg. Rectal temperature was recorded via a self-inserted rectal probe to a depth of ~ 10 cm beyond the anal sphincter (Grant Instruments Ltd., Cambridge, England). A heart rate monitor was attached (Polar Electro, Kempele, Finland) and a cannula inserted into an antecubital vein (Venfon 20G, Sweden) and kept patent with saline (0.9% Sodium Chloride BP). Participants then stood for 15 min and a resting blood sample was collected followed by resting T_rec_. Baseline cognitive tests were then administered.

On completion of the baseline tests, participants either entered the environmental chamber (~ 40 °C, 75% rh) to begin a period of dehydration via controlled passive hyperthermia or remained euhydrated, with the ingestion of water ad libitum, in a thermally neutral environment (~ 19 °C) for ~ 2 h. During the dehydration trials (HNF, MNF), participants were initially dehydrated for 28 ± 25 and 66 ± 44 min for the hot and moderate trials, respectively. Participants remained in the environmental chamber in a semi-recumbent posture to elicit a mass loss based on measurements obtained during familiarisation (1–1.5% of initial BM). The remaining mass loss was achieved through sweat loss during the FHITP. To increase sweat rate, participants wore non-permeable water proofs for the entire duration of the protocol. To verify the loss of body water and to minimise a rise in T_rec_, participants were removed from the environmental chamber every 20 min for measurement of nude body mass. Participants received no water during the dehydration phase. This was to ensure participants achieved a standard 2% loss in body mass across dehydration trials. On completion, participants returned to a thermally neutral environment (~ 19 °C) until T_rec_ returned to resting values (~ 1 h). Within this time period, skin temperature and thermal sensation had both returned to baseline levels.

Participants next entered the environmental chamber maintained under moderate (16 ± 3 °C, 53 ± 2% rh) or hot (33.3 ± 0.1 °C, 59 ± 1%) environmental conditions. After 15 min of exposure, baseline T_rec_ and heart rate were recorded. Participants completed a warm-up consisting of jogging at a self-selected pace which was the same for all trials, for ~ 10 min before the commencement of the 50 min FHITP. During the trial, participants were informed of the type of exercise required at each stage by following a slide show generated in PowerPoint (Microsoft Windows XP, Microsoft Corporation, USA). During the fluid trials, water was available ad libitum and the amount consumed was recorded.

Heart rate was recorded continuously throughout the trial (sampling every 5 s). Rectal temperature, rating of perceived exertion [57] and perceived thirst (9 point scale ranging from not thirsty to very, very thirst) were measured every 10 min. On completion of the intermittent exercise protocol, participants remained in the chamber to repeat the cognitive test battery (test 2) within 5 min of the cessation of exercise. After completing test 2 (~ 15 min) participants were removed from the chamber and towel-dried before recording a dry nude post-exercise body mass from which sweat loss was calculated and adjusted for fluid consumed and any urinary losses.

### Blood sampling and analysis

Twenty ml blood samples were taken at rest, at the start of the FHITP, at half-time during the FHITP, at the end of the FHITP and after the final cognitive test battery. One millilitre of blood was immediately analysed for blood glucose and lactate (Yellow Springs Instrument 2300 STAT plus, Yellow Springs Instruments Inc., Ohio, USA). Haematocrit and haemoglobin were analysed in triplicate via microcentrifugion (Hawksley, Hawklsey and Sons Ltd., Sussex, UK) and B-hemoglobin photometry (HemoCue, HemoCue AB, Ängelholm, Sweden), respectively. Changes in plasma volume were calculated using the method of Dill and Costill [58]. The remaining blood was dispensed into pre-chilled EDTA KE tubes (Sarstedt Ltd., Leicester, UK) and serum tubes (Sarstedt Ltd., Leicester, UK). Samples were centrifuged at 3000 g for 15 min at 4 °C before the plasma and serum was separated and divided into aliquots. One aliquot was immediately used for the determination of serum osmolality (in triplicate) via freezing-point depression (Osmomat 030, Genotec, Berlin, Germany). The remaining aliquots were then initially frozen at − 20 °C before being stored at − 80 °C for later analysis. Serum concentrations of progesterone were determined via enzyme-linked immunosorbent assays (DRG Instruments GmbH, Marburg, Germany). The intra-assay variation (CV) for progesterone was 12.3%.

### Statistical analyses

Descriptive data is reported as mean ± (SD) and are based on a participant population of eight unless otherwise stated. Rectal temperature, heart rate, perceptual responses, blood lactate, blood glucose and serum osmolality were analysed using a three-way (condition x fluid x time) repeated measures ANOVA to evaluate differences between and within conditions. Following a significant F value Tukey’s post hoc tests were conducted to identify differences in the way participants responded to the FHITP in the four conditions. Environmental conditions, baseline body mass, urine osmolality, estimated sweat loss, estimated plasma volume and serum progesterone were analysed using a two-way (condition x fluid) repeated measures ANOVA with Tukey tests where necessary. A students *t*-test was also used where appropriate. Cognitive test data were analysed using a four-way (condition x fluid x time x test level) repeated measures ANOVA. Following a significant F value Tukey’s post hoc tests were conducted to identify differences. Due to the complexity of the analysis of the cognitive function data, only significant findings are presented. Violations of sphericity were adjusted for using the Greenhouse Geisser adjustment when appropriate. In line with the recommendations of Cohen (1992) a priori power analysis was completed with a power of 0.8, an alpha level of 0.05 and effect size of 0.4 which estimated a sample size of 8 would be sufficient to detect a difference (G Power 3.1 [59]). The effect size (Cohen’s *d*) of all significant differences were calculated using trial pairings and interpreted using the following thresholds: < 0.2 = trivial effect; 0.2–0.5 = small effect; 0.5–0.8 = moderate effect and > 0.8 = large effect [60]. For all analyses, significance was set at the 5% level.

## Results

### Environmental conditions

Temperatures were higher in the hot trials than the moderate trials (HF = 33.24 ± 0.10 °C, HNF = 33.30 ± 0.03 °C vs. MF = 16.29 ± 0.03 °C, MNF = 16.45 ± 0.03 °C; main effect condition, *P* < 0.001) with no difference between fluid trials (main effect fluid, *P* = 0.219). Relative humidity was greater in the hot trials (HF = 58 ± 1%, HNF = 59 ± 1% vs. MF = 53 ± 2%, MNF = 53 ± 2%, main effect condition, *P* = 0.010).

### Cognitive performance

Data from the cognitive function tests are presented in Table 1.

**Table 1:** Mean ± SD (range) response time and accuracy for the Stroop, Visual Search and Sternberg cognitive function tests

			Response Time (ms)				Accuracy (%)			
Test	Level		Hot		Moderate		Hot		Moderate	
			Fluid	No Fluid	Fluid	No Fluid	Fluid	No Fluid	Fluid	No Fluid
Stroop	Baseline	Pre-exercise	604 ± 90 (473–766)	599 ± 70 (509–739)	606 ± 63 (487–685)	573 ± 64 (497–682)	95.6 ± 3.2 (90–100)	98.1 ± 3.7 (90–100)	98.1 ± 2.6 (95–100)	96.9 ± 3.7 (90–100)
		Post-exercise	585 ± 94 (472–695)	572 ± 87 (461–694)	579 ± 68 (472–676)	577 ± 81 (496–746)	98.8 ± 2.3 (95–100)	95.6 ± 3.2 (90–100)	98.8 ± 2.3 (95–100)	98.1 ± 3.7 (90–100)
	Complex	Pre-exercise	772 ± 167 (599–1052)	757 ± 144 (587–1031)	722 ± 97 (589–895)	724 ± 103 (556–837)	97.8 ± 2.3 (94–100)	95.5 ± 3.7 (90–100)	96.3 ± 4.7 (88–100)	97.5 ± 2.8 (92–100)
		Post-exercise	697 ± 131 (555–854)	688 ± 96 (578–857)	698 ± 96 (571–883)	708 ± 91 (628–876)	97.0 ± 2.4 (94–100)	96.8 ± 4.1 (88–100)	95.8 ± 4.5 (88–100)	96.5 ± 2.1 (92–98)
			Condition * time interaction, *p* = 0.080 Post-hoc: hot pre-exercise > post-exercise (*p* = 0.014)							
Visual Search	Baseline	Pre-exercise	496 ± 16 (471–516)	505 ± 23 (482–549)	500 ± 15 (475–528)	508 ± 20 (483–545)	98.9 ± 3.2 (90.9–100)	99.4 ± 1.7 (95.2–100)	99.4 ± 1.7 (95.2–100)	99.4 ± 1.7 (95.2–100)
		Post-exercise	481 ± 22 (464–535)	498 ± 39 (463–585)	515 ± 17 (493–540)	496 ± 9 (486–510)	99.4 ± 1.7 (95.2–100)	98.8 ± 2.2 (95.2–100)	100.0 ± 0.0 (100–100)	99.4 ± 1.7 (95.2–100)
	Complex	Pre-exercise	1932 ± 288 (1496–2354)	2060 ± 499 (1222–2810)	2010 ± 411 (1326–2733)	2174 ± 540 (1284–2787)	99.5 ± 1.3 (96.2–100)	98.2 ± 3.8 (89.3–100)	99.0 ± 2.0 (94.3–100)	98.8 ± 2.6 (92.6–100)
		Post-exercise	1847 ± 295 (1228–2054)	1926 ± 304 (1360–2246)	2115 ± 321 (1655–2754)	2119 ± 356 (1294–2409)	99.3 ± 2.0 (94.3–100)	95.3 ± 6.5 (90.9–100)	99.3 ± 1.4 (96.2–100)	99.8 ± 0.7 (98–100)
			Condition * test level interaction, *p* = 0.005 Post-hoc: complex level hot < moderate (*p* = 0.001)							
Sternberg	1 item	Pre-exercise	401 ± 51 (343–492)	434 ± 59 (371–571)	402 ± 43 (347–475)	393 ± 44 (337–465)	98.4 ± 4.4 (87.5–100)	94.5 ± 6.2 (81.3–100)	92.9 ± 7.0 (81.3–100)	95.3 ± 8.7 (87.5–100)
		Post-exercise	390 ± 36 (336–448)	372 ± 33 (332–433)	397 ± 33 (348–449)	394 ± 41 (333–447)	98.4 ± 2.9 (93.8–100)	94.5 ± 6.2 (81.3–100)	97.7 ± 4.7 (87.5–100)	97.7 ± 4.7 (87.5–100)
	3 item	Pre-exercise	468 ± 48 (398–520)	475 ± 52 (392–547)	494 ± 40 (431–558)	463 ± 68 (376–546)	96.9 ± 2.4 (93.8–100)	97.3 ± 3.5 (90.6–100)	94.9 ± 4.4 (87.5–100)	95.7 ± 3.7 (90.6–100)
		Post-exercise	435 ± 53 (365–515)	451 ± 58 (397–563)	468 ± 43 (414–518)	467 ± 46 (397–530)	96.9 ± 3.7 (90.6–100)	97.7 ± 2.2 (93.8–100)	96.9 ± 1.7 (93.8–100)	96.9 ± 1.7 (93.8–100)
	5 item	Pre-exercise	564 ± 76 (480–678)	523 ± 64 (451–639)	555 ± 33 (510–605)	564 ± 85 (473–691)	96.5 ± 3.1 (90.6–100)	95.7 ± 6.7 (81.3–100)	95.7 ± 3.7 (90.6–100)	97.3 ± 2.0 (93.8–100)
		Post-exercise	528 ± 76 (447–663)	555 ± 33 (510–605)	534 ± 52 (474–605)	549 ± 67 (441–648)	96.1 ± 2.8 (90.6–100)	93.4 ± 6.6 (78.1–100)	96.9 ± 4.4 (87.5–100)	97.3 ± 1.1 (96.9–100)
			Main effect of condition, *p* = 0.024 Post-hoc: hot < moderate (*p* = 0.024) Main effect of time, *p* = 0.007 Post-hoc: pre-exercise > post-exercise (*p* = 0.007)				Condition * time interaction, *p* = 0.004 Post-hoc: moderate pre-exercise < post-exercise (*p* = 0.044)			

### Stroop test

*Response Times:* There was no effect of condition or fluid on response times on the Stroop test (all *P* > 0.05). However, there was a tendency for quicker response times following exercise, but only in the heat (Hot: pre-exercise 683 ms, post-exercise 636 ms (*p* = 0.014; *d* = 0.39); Moderate: pre-exercise 656 ms, post-exercise 640 ms (*p* = 0.507; *d* = 0.16); condition x time interaction, *p* = 0.080).

*Accuracy:* There was no effect of condition, fluid or time on accuracy on either the baseline or colour interference levels of the Stroop test (all *p* > 0.05).

### Visual search

*Response Times:* Response times on the visual search test were quicker in the heat, but this was only the case on the complex level. On the baseline level, response times were similar regardless of environmental condition (Baseline level: hot 495 ms, moderate 505 ms (*p* = 0.982; *d = 0.49*); Complex level: hot 1941 ms, moderate 2104 ms (*p* = 0.001; *d* = 0.47); condition x test level interaction, *p* = 0.005).

*Accuracy:* There was no effect of condition, fluid or time on accuracy on either the baseline or complex levels of the visual search test (all *p* > 0.05).

### Sternberg paradigm

*Response Times:* Response times on the Sternberg paradigm were quicker in the hot conditions (hot: 463 ms, moderate: 473 ms; main effect of condition, *p* = 0.024; *d =* 0.13). Furthermore, response times were quicker following exercise, though this was irrespective of condition and fluid (pre-exercise: 478 ms, post-exercise: 459 ms; main effect of time, *p* = 0.007; *d* = 0.26).

*Accuracy:* Participants achieved a greater percentage of correct responses following exercise, though this was only the case in the moderate conditions. In the heat, accuracy was unchanged following exercise (Hot: pre-exercise 96.8%, post-exercise 96.3% (*p* = 0.613; *d* = 0.12); Moderate: pre-exercise 95.4%, post-exercise 97.3% (*p* = 0.044; *d = 0.46*); condition x time interaction, *p* = 0.004).

### Body fluid balance

There was no main effect differences in baseline body mass between conditions (*P* = 0.896) or fluid trials (*P* = 0.986; HF = 62.84 ± 6.41 kg, HNF = 62.84 ± 6.06 kg, MF = 62.84 ± 6.11 kg, MNF = 62.83 ± 6.49 kg). There was no main effect difference between conditions (*P* = 0.052) in percentage change in body mass from baseline (Rest) to the end of the protocol (Post-Cog). However, body mass loss was considerably greater in the dehydration trials (main effect fluid, *P* < 0.001, *d = 4.35*; HF = 0.5 ± 0.5% (range: 0.9 to + 0.2%), HNF = 2.6 ± 0.6% (range: 1.9 to 2.8%), MF = 0.5 ± 0.6% (range: 1.5 to + 0.3%), MNF = 2.1 ± 0.4% (range: 1.6 to 2.6%).

Baseline *U*_osm_ was less than 800 mOsm^.^kg^− 1^ for all trials (HF = 687 ± 115 mOsm^.^kg^− 1^, HNF = 642 ± 161 mOsm^.^kg^− 1^, MF = 698 ± 165 mOsm^.^kg^− 1^, MNF = 632 ± 155 mOsm^.^kg^− 1^). No difference existed between conditions (*P* = 0.259) or fluid trials (*P* = 0.779). Baseline *S*_osm_ was similar across all trials (HF = 286 ± 3 mOsm^.^kg^− 1^, HNF = 287 ± 4 mOsm^.^kg^− 1^, MF = 286 ± 3 mOsm^.^kg^− 1^, MNF = 287 ± 5 mOsm^.^kg^− 1^). Serum osmolality increased over time (*P* < 0.001), was higher when conditions were hot (main effect condition, *P* = 0.015) and when participants were dehydrated (main effect fluid, *P* < 0.001). Also, when the four trials were compared Post-Cog, *S*_osm_ was higher when water intake was restricted during the FHITP (HF = 288 ± 2 mOsm^.^kg^− 1^, HNF = 300 ± 2 mOsm^.^kg^− 1^, MF = 288 ± 2 mOsm^.^kg^− 1^, MNF = 297 ± 4 mOsm^.^kg^− 1^, interaction fluid x time, *P* < 0.001). Estimated changes in plasma volume showed a main effect of time (*P* = 0.031) and decreased significantly when conditions were hot (main effect condition, *P* = 0.012) and when fluid was restricted (HF = − 2.4 ± 3.1%, HNF = − 8.1 ± 3.6%, MF = − 1.7 ± 2.8%, MNF = − 7.8 ± 3.7%; main effect fluid, *P* = 0.021).

Estimated sweat rate during the FHITP was significantly higher when conditions were hot (main effect condition, *P* < 0.001) but no difference was observed between fluid trials (main effect fluid, *P* = 0.871; HF = 1.41 ± 0.18 l.h^− 1^, HNF = 1.53 ± 0.31 l.h^− 1^, MF = 0.89 ± 0.36 l.h^− 1^, MNF = 0.81 ± 0.31 h.l^− 1^). Participants consumed nearly twice as much water during the FHITP in the hot trial compared to the moderate trial (922 ± 237 ml, 435 ± 257 ml; main effect condition, *P* < 0.001).

### Heart rate and rectal temperature

**Fig. 1:**
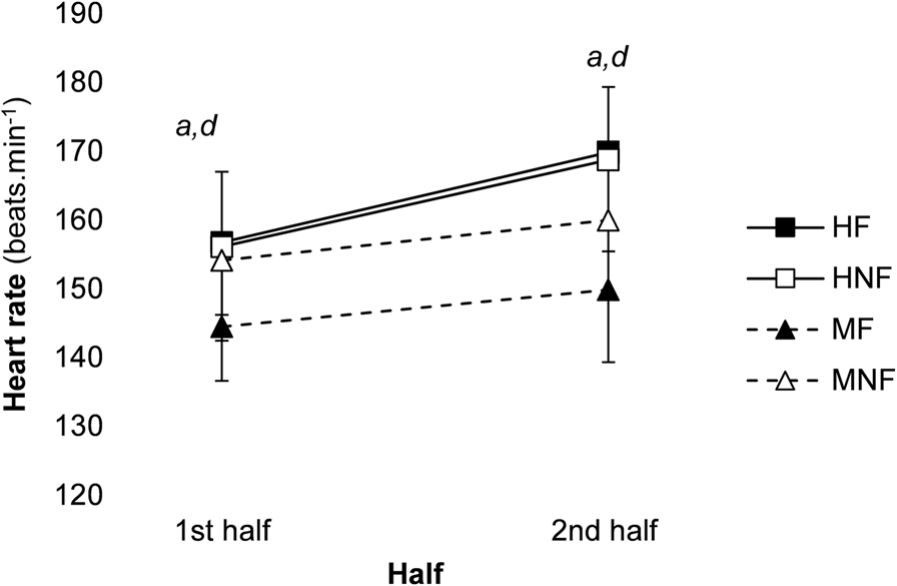
The 1^st^ half and 2^nd^ half heart rate data (mean ± SD) observed during the FHITP. (HF: hot environment with fluid; HNF; hot environment no fluid; MF; moderate environment with fluid; MNF: moderate environment no fluid). Main effect condition (*P* = 0.001), hydration state (*P* = 0.020), time (*P* = 0.001) and condition x time interaction (*P* = 0.005). *a* = *P* < 0.05 between HF and MF, *d = P* < 0.05 between HNF and MF

Figure 1 shows heart rate during the 1^st^ half and 2^nd^ half of the FHITP. Heart rate significantly increased over time from the 1^st^ half to the 2^nd^ half (main effect time, *P* < 0.001), was significantly higher in the heat (main effect condition, *P* = 0.001) and when water was restricted (main effect fluid, *P* = 0.020). When all four trials were compared in the 1st half and the 2nd half of the FHITP, heart rate was higher when the conditions were hot (interaction condition x time, *P* = 0.005).

**Fig. 2:**
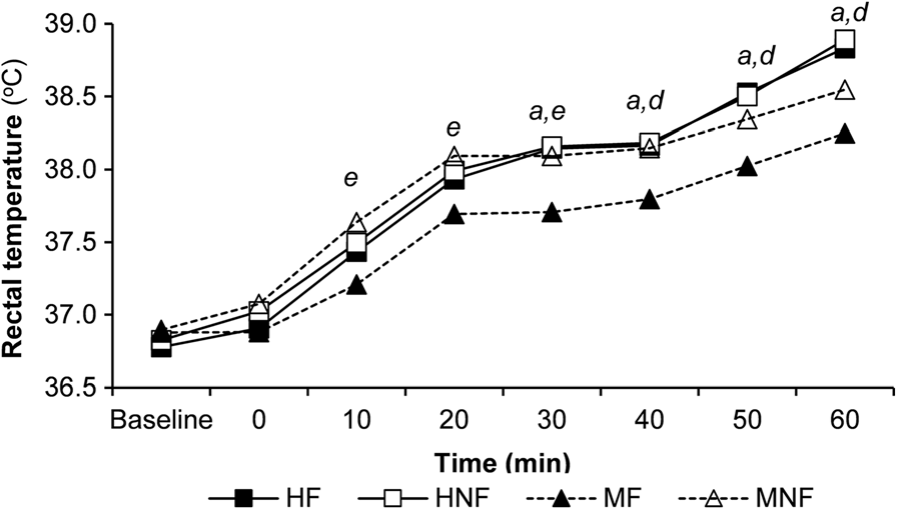
The mean (± SD) rectal temperature observed during the FHITP. (HF: hot environment with fluid; HNF; hot environment no fluid; MF; moderate environment with fluid; MNF: moderate environment no fluid). For clarity SD is not shown. SD for HF = ± 0.4; HNF = ± 0.4; MF = ± 0.4; MNF = ± 0.3. Main effect condition (*P* = 0.029), time (*P* < 0.001) and condition x time interaction (*P* < 0.001). *a* = *P* < 0.05 between HF and MF, *d = P* < 0.05 between HNF and MF, *e = P* < 0.05 between MF and MNF

Rectal temperature increased over time (main effect time, *P* < 0.001; Fig. 2), was higher when conditions were hot (main effect condition, *P* = 0.029) with a significant interaction effect for condition x time (*P* < 0.001). When all four trials were compared at rest and at 0 min no significant differences were observed (*P* > 0.05) indicating sufficient recovery between the period of passive hyperthermia and the start of the FHITP.

### Perceptual responses

**Fig. 3:**
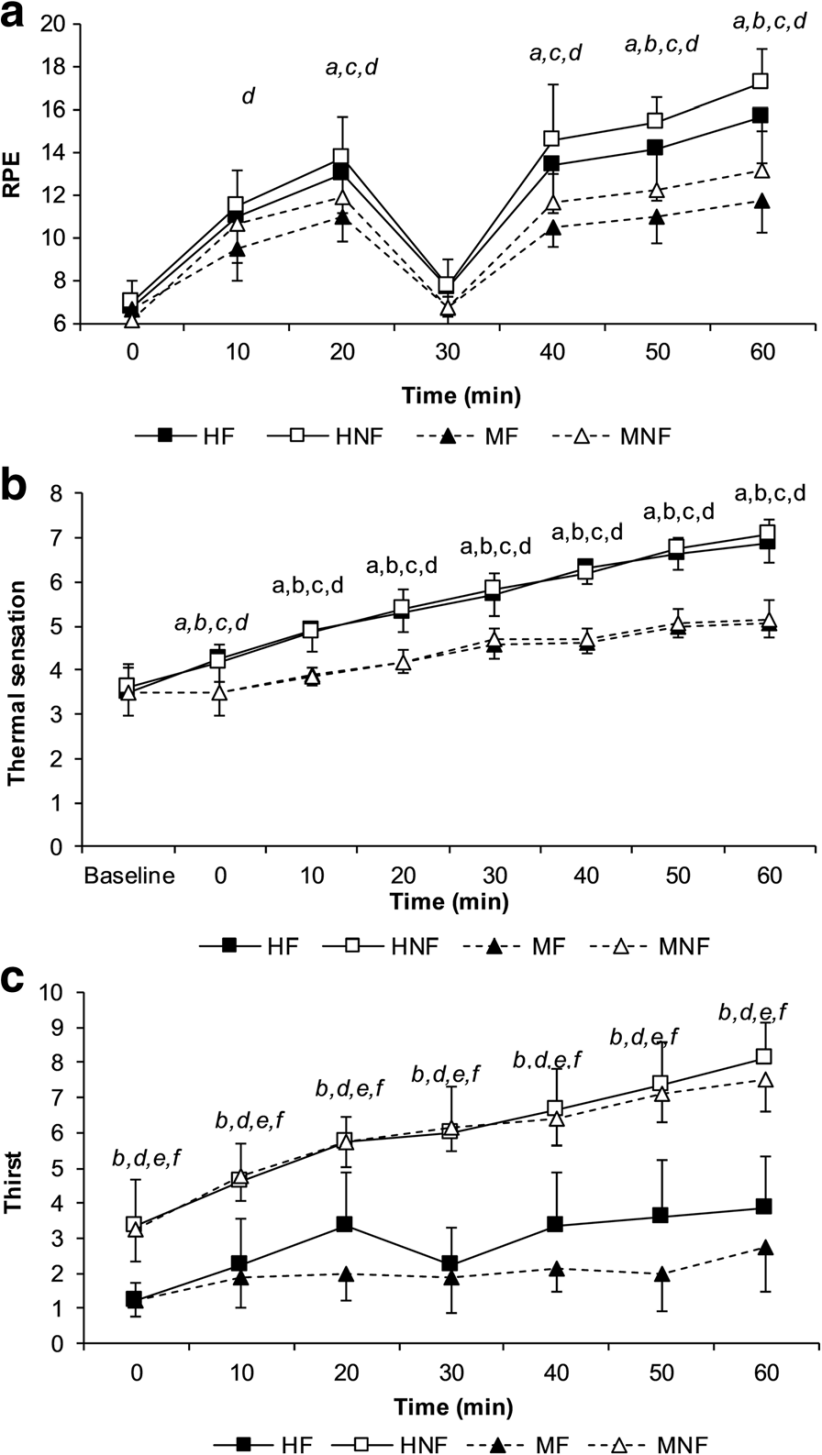
The mean (± SD) rating of perceived exertion (panel **a**), thermal sensation rating (panel **b**) and thirst sensation (panel **c**) observed during the FHITP. (HF: hot environment with fluid; HNF; hot environment no fluid; MF; moderate environment with fluid; MNF: moderate environment no fluid). RPE: Main effect condition (*P* < 0.001), fluid (*P* = 0.003), time (*P* < 0.001) and condition x time interaction (*P* < 0.001) and fluid x time interaction (*P* = 0.002). Thermal sensation: Main effect condition (*P* < 0.001), time (*P* < 0.001) and condition x time interaction (*P* < 0.001). Thirst sensation: Main effect fluid (*P* < 0.001), time (*P* < 0.001) and fluid x time interaction (*P* < 0.001) and condition x fluid x time interaction (*P* = 0.018). *a* = *P* < 0.05 between HF and MF, b = P < 0.05 between HF and MNF, c = *P* < 0.05 between HNF and MNF, *d = P* < 0.05 between HNF and MF, *e* = *P* < 0.05 between MF and MNF, *f* = P < 0.05 between HF and HNF

Rating of perceived exertion (Fig. 3) and thermal sensation (Fig. 3) increased through each half of the FHITP (main effect time, *P* < 0.001) and were greater when the conditions were hot (main effect condition, *P* < 0.001; interaction condition x time, *P* < 0.001). RPE was greater when water was restricted during the FHITP (main effect fluid, *P* = 0.003; interaction fluid x time, *P* = 0.002).

Thirst sensation increased throughout each half of the FHITP (main effect time, *P* < 0.001). There was no difference between conditions (*P* = 0.107) however, thirst sensation was significantly higher when water was restricted (main effect fluid, *P* < 0.001; interaction effect fluid x time, *P* < 0.001; Fig. 3). When all trials were compared, thirst sensation was higher at all time points during the dehydration trials in both the hot and moderate conditions (interaction effect condition x fluid x time, *P* = 0.018).

### Blood data

Resting serum progesterone concentration was similar between trials (HF = 1.46 ± 0.96 nmol^− 1^, HNF = 1.33 ± 0.91 nmol^− 1^, MF = 1.68 ± 1.39 nmol^− 1^, MNF = 1.53 ± 0.97 nmol^− 1^; main effect condition, *P* = 0.260; main effect fluid, *P* = 0.231) and indicated all participants undertook each trial during the follicular phase of the menstrual cycle and at the same time during the pill cycle.

**Fig. 4:**
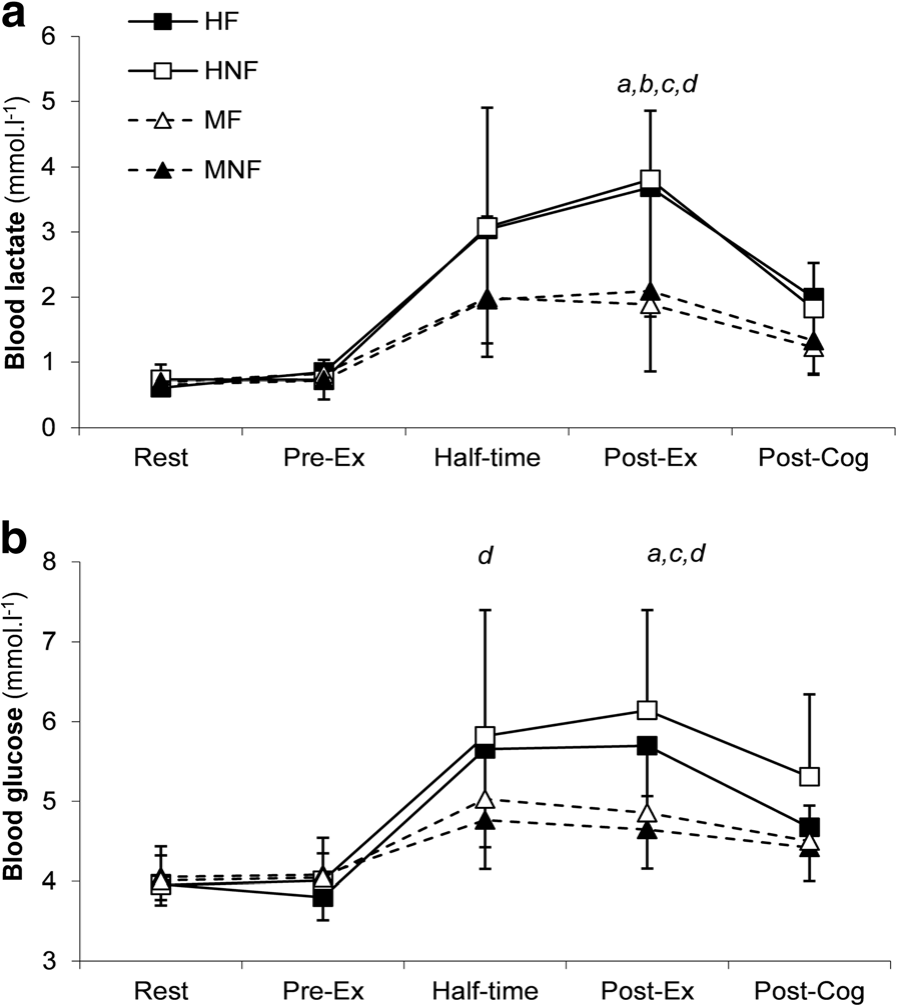
The mean (± SD) blood lactate (panel **a**) and glucose (panel **b**) concentration during the FHITP. (HF: hot environment with fluid; HNF; hot environment no fluid; MF; moderate environment with fluid; MNF: moderate environment no fluid). Lactate: Main effect condition (*P* = 0.040), time (*P* < 0.001) and condition x time interaction (*P* < 0.001). Glucose: Main effect condition (*P* = 0.025), time (*P* < 0.001) and condition x time interaction (*P* < 0.001). *a* = *P* < 0.05 between HF and MF, b = *P* < 0.05 between HF and MNF, c = *P* < 0.05 between HNF and MNF, *d* = *P* < 0.05 between HNF and MF

Blood lactate concentration increased over time during the FHITP (main effect time, *P* < 0.001) and was greater when the conditions were hot (main effect condition, *P* = 0.040; Fig. 4). When all four trials were compared, blood lactate was greater at half-time and post-ex in the heat (interaction condition x time, *P* < 0.001). Blood glucose increased over time during the FHITP (main effect time, *P* < 0.001) and was significantly greater when the conditions were hot (main effect condition, *P* = 0.025; interaction effect condition x time, *P* < 0.001; Fig. 4).

## Discussion

The aim of this study was to investigate the effect of heat stress and dehydration on cognitive performance following field hockey-specific intermittent exercise. The novel findings of the present study were that 1) hot environmental conditions improved response times during executive function, complex visuo-motor and serial working memory tasks, 2) intermittent exercise, closely reflecting the physiological demands of field hockey match-play, improved response time regardless of the environmental conditions or hydration status of the participant, 3) intermittent exercise in moderate conditions improved serial working memory accuracy and 4) cognitive function was unaffected by ~ 2% dehydration following intermittent exercise.

The cognitive function tests employed in the present study, assessed a variety of processes and skills. Frontal lobe function was assessed using an executive function task, specifically the Stroop test, which required the inhibition of an automated response [61]. The visual search test assessed visuo-motor response times and is predominantly an occipital lobe function test [53], while the Sternberg test assessed frontal lobe function using a serial working memory assessment [56]. During the game of field hockey, successful skilled performance has a substantial cognitive requirement [1], which may be susceptible to a number of physiological and environmental factors. The post-exercise body mass losses incurred during the HNF (2.3%) and MNF (2.1%) trials are similar to post-match body mass losses observed during competitive match-play [3] and are indicative of the threshold hypothesised to be sufficient to degrade cognitive performance [26–28]. Therefore, any changes in cognitive performance observed in the present study may reflect those during competitive play, and have application to field hockey skill performance in the competition setting.

The findings of the present study suggesting that dehydration does not impact upon cognitive function are in agreement with the findings of Marino and Serwah [30], who found no change in choice reaction time after fluid was restricted during 90 min of exercise in a warm, humid environment (31 °C, 63% rh). The authors concluded that fluid ingestion was not as important for decision-making as it is for physical performance [30]. A number of other studies have demonstrated similar findings, whereby dehydration has not influenced cognitive performance [62, 63], thus suggesting that hypohydration up to 2% body mass loss does not effect cognitive function. In the present study the range of dehydration was 1.6 to 2.8% demonstrating the individual variability you would see in the field. This is primarily related to the ad libitum drinking employed in the study design, which is reflective of on the field practise. This individual variation may impact on the findings presented at a group level.

Results obtained from studies in which dehydration was induced by exercise often differ in response when cognitive performance is assessed either during, immediately following exercise or after a substantial delay. Grego et al. [34] assessed cognitive performance during and immediately after 3 h of continuous cycling with restricted fluid intake. Interestingly, despite body mass losses of ~ 2% and ~ 4% in the fluid and no fluid trial, cognitive performance did not differ between trials. It is possible that dehydration exhibits less of an effect on cognitive performance during and immediately after exercise than after a delay. Another possible explanation may be that moderate dehydration only results in performance decrements when perceived discomfort increases as a result of the initiation of the thirst response. Although there are physiological mechanisms, which affect water intake, psychological factors and habit are also heavily influential. MacLeod and Sunderland [3] investigated hydration habits of elite female hockey plays during match-play and reported body mass losses of ~ 2%. Therefore, elite players may be familiar with the levels of dehydration employed in the present study and thus the level of hypohydration or feelings of fatigue induced as a result of the exercise intensity employed were insufficient to elicit a decrement in cognitive function, whereas this may be more of an issue in novice athletes. Furthermore, it is also possible that due to the positive effect of exercise on cognitive function [40], the potential detriment in cognition as a result of dehydration may be masked by the facilitating effects of exercise. The fluid restriction method of inducing dehydration has shown varying results, with some showing no difference in performance [64], however the majority of studies demonstrate a decrement in cognitive performance [65–67]. Thus the dehydration method employed and the training status of the athletes and their experience of dehydration appear to interact to determine whether cognitive function is altered during or after exercise in the heat.

An alternative theory is that participants with a high skill level are better able to withstand the effects of heat stress and exercise stress [21, 68]. Tasks that are familiar to the participant or are autonomous in nature are less susceptible to interference between stimulus and response. The participants in the present study were elite performers, familiar with the exercise demands and highly motivated individuals. As such, it seems plausible that increased motivation and attention maintained or improved performance on a number of tasks and heat stress increased arousal to help overcome any possible performance decrements as a result of dehydration. High level athletes are thought to be able to withstand higher levels of exercise stress, enabling greater attentional breadth, which may also enable better responses to dehydration [68].

The maintenance of cognitive function observed in the present study has been reported elsewhere in the literature [19] and may be due to an increase in arousal due to the activation of thermoregulatory mechanisms [23]. The improved response times during the executive function, complex visuo-motor and serial working memory tasks in the heat are reflective of an increase in motor nerve conduction velocity related to the higher core temperature response in the heat. Simmons et al. [20] established that when heat strain is reduced with the implementation of a head cooling device, cognitive performance remains unchanged, therefore suggesting that a high core temperature is the limiting factor of cognitive performance [20]. However, neck cooling has been shown to improve more complex cognitive tasks despite high core temperatures [69]. It should be noted that, although rectal temperature was significantly higher during the FHITP performed in hot environmental conditions, it did not reach the critical level (> 39 °C) suggested by Hancock [21] beyond which a decrease in cognitive performance is likely to occur.

Exercise alone appears to result in a small but positive effect on cognition, although many factors such as the intensity and duration of exercise may mediate this relationship [40]. In addition to this, fitness and psychological wellbeing can also result in variability in the influence of exercise on cognition, with the influence of an acute bout of exercise on cognition being partly determined by the fitness level of participants [40, 41]. In the present study, the FHITP appeared to induce sufficient arousal to increase speed of response on all cognitive tests, regardless of hydration status or heat stress, with no observed interaction with task complexity. Response time is a useful variable when assessing cognitive performance and is relevant in a number of sports situations whereby players are required to make various reactions to a ball or other players during match-play. Tomporowski [70] suggests that peripheral sensorimotor improvements, such as motivation, could be responsible for the observed changes in performance in the present study, rather than any significant physiological changes. As such, expectancy effects may have influenced results as previously suggested by Tomporowski and Ellis [71]. An unconscious decision may have been made to overcome the possible effects of fatigue. Highly trained individuals who frequently engage in high-intensity exercise and are highly motivated are likely to expect a beneficial effect of exercise and are able to exert additional effort to the task.

Research conducted to date has predominantly focussed on the influence of steady-state exercise on cognitive performance and not sport-specific intermittent exercise [35, 39]. Marriott et al. [72] also reported improvements in decision-making performance in skilful players following 90 min of treadmill running at a heart rate intensity corresponding to soccer match-play. In comparison with low-skilled soccer players, the authors reported that skilful players had the ability to maintain attentional focus and offset the feelings of fatigue. The authors hypothesised that the positive effect of exercise on decision-making was due to optimal arousal and narrowing of attention, thereby eliminating irrelevant cues [72]. In contrast, in research simulating the activity of goal line officials, cognitive function was impaired at 90 min [43], whereas in referees, cognitive performance was unchanged [42]. Hogervorst et al. [39] employed a similar cognitive testing battery as the present study (Stroop test and visual search test) reporting improvements in reaction-time after high intensity cycling for simple and complex tasks. In particular, performance on the complex level of the Stroop test improved with no observed speed-accuracy trade-off or increased perceived effort. As with the present study, this suggests that there appears to be no relationship between physical fatigue and mental fatigue. However, it may be that the exercise performed in the current study was not sufficient to induce significant fatigue. Due to the timing of the cognitive tests, heart rate and rectal temperature were still elevated above pre-exercise levels, which is indicative of increased arousal and increased activation of the central nervous system. More recently Tsukamoto et al. [73] demonstrated an improvement in executive function immediately, and 40 min following 4 repeated bouts of 4 min at 90% of peak VO_2_ of cycle exercise. However, when the 4 bouts were repeated following a 60 min rest period, the improvement in executive function could only be maintained for 10 min on completion of the protocol. Therefore this study suggests that repeated bouts of high intensity, sport-specific exercise, may have a dampening effect on the improvement in cognitive function [73]. Clearly therefore the exercise intensity, training status and familiarity with the exercise type, and level of fatigue interact and therefore effect cognitive function in potentially different and complex ways.

In addition, recent research, has demonstrated that complex cognitive function tasks can be affected by changes in skin temperature and thermal sensation without concomitant rises in core temperature during passive heat exposure [74]. Similarly, during exercise in the heat, improved accuracy during complex tests has been demonstrated with neck cooling which resulted in a lower thermal sensation, despite similar high core temperatures (> 39.5 °C) [69]. This research suggests that skin temperatures and thermal sensation also play an important role in cognitive function when core temperature is similar. In the present study, skin temperature, thermal sensation and core temperature were all higher in the heat when cognitive performance was improved. Thus additional research would be required to see if cognitive performance could be further enhanced through, for example, improving thermal sensation.

### Experimental limitations and future directions

There are several factors that should be considered when interpreting the findings of the present study. Participants were aerobically fit, elite female field hockey players who trained on a regular basis and were familiar with the stressor. It remains unclear whether the results obtained from the present participants generalise to men or to less skilled or trained players or umpires [75]. Further, the study was conducted on a relatively small sample size of eight participants, although given the elite status of the sample, its size is almost inevitably limited. However, it has to be acknowledged that the failure to detect any significant changes as a result of dehydration may reflect a lack of statistical power, but, several other studies also report no effect of dehydration on cognitive function [29–31].

The FHITP employed in the current study was designed to replicate the demands of field hockey. The total distance and number of sprints completed and heart rate response are reflective of those seen in International and National league hockey [50, 51]. The addition of lunging movements, which are integral to field hockey and the carrying of the stick improved the validity of the protocol. However, side-wards and backwards running, channelling and the asymmetric position adopted in hockey will increase the demands of the activity and cannot be replicated on a treadmill. Therefore in the FHITP the time spent doing high speed running (16%) is greater than that observed in motion analysis field hockey research [50, 51], in an attempt to account for the absence of these physiologically demanding movements in the protocol. The movement demands of the FHITP was the same in all trials to ensure the effects of heat and hydration on cognitive function could be examined. Clearly, it could be argued that this does not reflect fatigue seen during a match or in a hot environment and some authors have used a non-motorised treadmill in research investigating the effect of team sport exercise on cognitive function [76]. However, non-motorised treadmills have a high belt resistance which decreases maximal running speed by up to 20% [77], although it has been suggested that a gradient be applied to counteract this resistance [78]. Due to the intrinsic resistance of a non-motorised treadmill belt and the increased probability of participant variability in terms of motivation [79], the decision was taken to employ a motorised treadmill in the present study.

The present study investigated the group mean responses to high intensity intermittent exercise in the heat with and without dehydration upon cognitive function. Individual variability in terms of hydration levels, tolerance to dehydration, high core temperatures and thermal sensation and thus their impact on cognitive function was not considered. Future research, using elite players, may assess this variation between individuals, employing large sample sizes.

### Practical applications

The findings of the present study suggest that elite field hockey players are able to cope with the expected deleterious effects elicited by the stressors of heat and dehydration (~ 2%), hence maintaining and even enhancing their cognitive function. Players would be advised to continue to try to maintain euhydration during matches as individual responses may vary and > 2% dehydration may be detrimental. The findings of the present study emphasise the importance of utilising elite level players, as the changes in their cognitive function may differ from their non-elite counterparts.

## Conclusions

The effects of dehydration and heat stress have been studied extensively in their own right. Less well understood, however, is the complex interaction of various environmental stressors, frequently encountered by our international team sports players, on cognitive performance. The findings of the present study are particularly poignant for players required to make rapid decisions in complex game situations under extreme environmental conditions. In conclusion, the FHITP improved generic response times on all cognition tests with no observed interaction with test accuracy. The accuracy of working memory improved following FHITP in moderate conditions regardless of hydration status. Hot environmental conditions improved response times during selective attention, complex visuo-motor and serial working memory tasks. Increased arousal and the availability of attentional cognitive resources, as a result of performing exercise in the heat may have enabled elite female field hockey players to improve response times during cognitive tasks. In conclusion, cognitive performance of elite female field hockey players is unaffected by ~ 2% dehydration following intermittent exercise in the heat.

## Abbreviations

BM: Body mass
FHITP: Field hockey intermittent treadmill protocol
HF: Hot fluid
HNF: Hot no fluid
MF: Moderate fluid
MNF: Moderate no fluid
T_rec_: Rectal temperature
